# MRI features of histological subtypes of thyroid cancer in comparison with CT findings: differentiation between anaplastic, poorly differentiated, and papillary thyroid carcinoma

**DOI:** 10.1007/s11604-024-01660-x

**Published:** 2024-09-18

**Authors:** Takahide Maeda, Hiroki Kato, Tomohiro Ando, Masaya Kawaguchi, Hirofumi Shibata, Takenori Ogawa, Yoshifumi Noda, Fuminori Hyodo, Masayuki Matsuo

**Affiliations:** 1 Department of Radiology, Gifu University, 1-1 Yanagido, Gifu, 501-1194 Japan; 2 Department of Otolaryngology, Gifu University, Gifu, Japan; 3 Department of Frontier Science for Imaging, Gifu University, Gifu, Japan; 4 Department of Pharmacology, School of Medicine, Gifu University, Gifu, Japan; 5 Center for One Medicine Innovative Translational Research (COMIT), Gifu University, Gifu, Japan

**Keywords:** Thyroid cancer, Anaplastic thyroid carcinoma, Poorly differentiated thyroid carcinoma, Papillary thyroid carcinoma, MRI

## Abstract

**Purpose:**

This study aimed to evaluate the MRI features of the main histological subtypes of thyroid cancer and enable differentiation between anaplastic thyroid carcinoma (ATC), poorly differentiated thyroid carcinoma (PDTC), and papillary thyroid carcinoma (PTC).

**Materials and methods:**

This study included 79 patients with histopathologically proven thyroid cancer (14 ATCs, 8 PDTCs, and 57 PTCs) who underwent neck MRI. MRI images were retrospectively reviewed and compared between the three pathologies.

**Results:**

The maximum diameter was larger in ATCs and PDTCs than in PTCs (65.2 mm and 38.4 mm vs. 26.0 mm, *p* < 0.01). The signal intensity ratio of the solid components on T2-weighted images (T2WIs) was higher in ATCs than in PTCs (1.13 vs. 0.89, *p* < 0.05). The predominant signal intensity of the solid components on T2WI exhibited hyperintensity relative to the spinal cord in ATCs more frequently than in PTCs (71% vs. 30%, *p* < 0.01), whereas hypointensity was more frequent in PTCs than in ATCs and PDTCs (60% vs. 0% and 13%, *p* < 0.01). Intratumoral ring-shaped hypointensity on T2WI was more frequent in ATCs than in PDTCs and PTCs (64% vs. 13% and 18%, *p* < 0.01). An ill-defined margin was more frequent in ATCs and PDTCs than in PTCs (93% and 63% vs. 25%, *p* < 0.01). Extrathyroidal extension, tracheal invasion, esophageal invasion, vascular invasion, and venous thrombosis were more frequently observed in ATCs than in PTCs (*p* < 0.05).

**Conclusions:**

MRI could characterize the differences between ATCs, PDTCs, and PTCs.

## Introduction

Thyroid cancer is the ninth most common type of cancer worldwide. Approximately 75% of patients with thyroid cancer are females, and the median age of diagnosis is in the early 50 s. Furthermore, this disease is the most common malignancy in the 16–33 age group [1].

Thyroid cancer can usually be classified into four major histological categories: (1) well-differentiated thyroid carcinoma (WDTC), which includes papillary thyroid carcinoma (PTC) and follicular thyroid carcinoma (FTC); (2) anaplastic thyroid carcinoma (ATC), which has a higher incidence of adjacent organ invasion and distant metastasis with worse prognosis; (3) poorly differentiated thyroid carcinoma (PDTC), which shows intermediate morphological features and biological behavior between WDTC and ATC; and (4) medullary thyroid carcinoma, which is derived from the parafollicular cell and is sometimes associated with multiple endocrine neoplasia type 2 [1, 2].

Accurate diagnosis of ATC and PDTC is essential because they are rare malignancies with a poor prognosis, thereby requiring prompt medical intervention [3, 4]. Therefore, it is clinically useful to determine the radiological features of ATC/PDTC that help distinguish these histological categories from other thyroid masses. Several previous studies demonstrated the characteristic CT features of ATC, including its relatively large size, dense calcification, intratumoral necrosis, organ invasion, vascular invasion, and nodal necrosis [5–9]. However, to date, no study has shown the MRI characteristics of each histological subtype of thyroid cancer. In addition, the differences in imaging findings between PDCT and other histological subtypes of thyroid cancer remain unclear. Hence, the present study intended to determine the MRI characteristics of the histological subtypes of thyroid cancer, thereby differentiating between ATC, PDTC, and PTC.

## Materials and methods

### Patients

The present study was approved by the human research committee of the institutional review board of our hospital and complied with the guidelines of the Health Insurance Portability and Accountability Act of 1996 and the Declaration of Helsinki. The requirement for informed consent was waived due to the retrospective nature of the study. Two-hundred and eighty-two patients with histopathologically proven thyroid cancer (ATC, PDTC, or PTC) were identified from the electronic medical record database of our hospital between December 2006 and June 2023. Among them, 203 patients who did not undergo neck MRI (7 ATCs, 5 PDTCs, and 191 PTCs) were excluded. In total, 79 patients (14 ATCs, 8 PDTCs, and 57 PTCs) were enrolled in this study.

### Imaging technique

MRI was performed for all patients using 1.5 Tesla MRI scanners (Intera Achieva 1.5 T Pulsar, Philips Healthcare, Best, the Netherlands, or Ingenia Prodiva 1.5 T CS, Philips Healthcare, Best, the Netherlands) or a 3.0 Tesla MRI scanner (Intera Achieva 3.0 T Quasar Dual, Philips Healthcare, Best, the Netherlands). All images were obtained at a section thickness of 3–4 mm with an intersection gap of 1 mm. Axial non-fat-suppressed T2-weighted images (T2WIs) and axial non-fat-suppressed T1-weighted images (T1WIs) were obtained from all patients. Axial short-tau inversion recovery single-shot spin-echo echo-planar diffusion-weighted images (DWIs) with b values of 0 and 1000 s/mm were obtained from 68 patients (11 ATCs, 7 PDTCs, and 50 PTCs). Axial fat-suppressed contrast-enhanced T1-weighted images (FS CE-T1WIs) were obtained from 46 patients (8 ATCs, 5 PDTCs, and 33 PTCs) after the intravenous injection of 0.1 mmol/kg of gadopentetate dimeglumine (Magnevist; Bayer HealthCare, Leverkusen, Germany) or gadobutrol (Gadavist; Bayer HealthCare).

CT imaging was performed for all patients using an 8-slice CT scanner (LightSpeed Ultra; GE Healthcare, Milwaukee, WI, USA), or 16-slice CT scanner (LightSpeed 16; GE Healthcare, Milwaukee, WI, USA), or 64-slice CT scanner (Brilliance 64; Philips, Best, the Netherlands or Discovery CT750 HD; GE Healthcare, Milwaukee, WI, USA), or 256-slice CT scanner (Revolution CT Apex Edition; GE Healthcare, Milwaukee, WI, USA). Unenhanced CT images were obtained from all patients. All transverse CT images were reconstructed with 2.5-mm section thickness and no overlap.

### Imaging assessment

Two radiologists (radiologists-1 and -2) with post-training experience in head-and-neck imaging of 24 and 10 years, respectively, reviewed all MRI images individually and randomly. The reviewers were unaware of any clinical information or pathological diagnosis. Any disagreement between the two reviewers was resolved through discussion until a consensus was reached.

For quantitative assessments, radiologist-1 measured the maximum tumor diameter of the whole lesion and the signal intensity (SI) of the solid components of the tumors. The SI of the solid components on T1WI, T2WI, and FS CE-T1WI was measured by using regions of interest (ROIs), which were carefully placed on the solid components as widely as possible in the slice showing the maximum diameter by referring to T2WI and/or FS CE-T1WI. The reviewers measured the SI of the spinal cord at the same level as the lesion by placing 10-mm diameter circle ROIs and calculating the tumor-to-spinal cord signal intensity ratio (SIR). The mean apparent diffusion coefficient (ADC) values of the solid components of the tumor were also measured on ADC maps by placing ROIs. When ring-shaped hypointensity was found within the tumor on T2WI, the reviewers measured the diameter and maximum thickness of the area.

For qualitative assessments, radiologists-1 and -2 evaluated tumor laterality (unilateral or bilateral) and the tumor margin (well-defined or ill-defined). On T2WI and T1WI, they qualitatively evaluated internal uniformity (homogeneous or heterogeneous) and the predominant SI of the solid components relative to the spinal cord (hyperintensity, isointensity, or hypointensity). The presence of hypointensity similar to the muscle on T2WI, intratumoral ring-shaped hypointensity on T2WI, and hyperintensity relative to the muscle on T1WI were also assessed. Furthermore, on FS CE-T1WI, both radiologists assessed internal uniformity (homogeneous or heterogeneous) and the presence of an unenhanced area. In addition, the presence of extrathyroidal extension, tracheal invasion, esophageal invasion, laryngeal invasion, vascular invasion, venous thrombosis, venous tumor thrombosis, and lymphadenopathy was assessed. Extrathyroidal extension included focal outer protrusion, irregular outer protrusion, surrounding fat stranding, or adjacent organ invasion. Tracheal, esophageal, laryngeal, and vascular invasions were defined as tracheal intraluminal invasion, esophageal intramuscular invasion, laryngeal soft-tissue or cartilage invasion, and solid soft-tissue contact > 180° between the tumor and vessel circumference, respectively. Lymphadenopathy was considered positive when the minimum diameter exceeded 10 mm or an intranodal cystic area was found.

In addition, the presence of calcification within the tumor was assessed on CT images. CT attenuations of calcification were defined as > 200 HU. If calcification was observed within the tumor, the number (single or multiple) and shape (nodular- and/or ring-shaped) of calcification were determined. In the cases with ring-shaped hypointensity on T2WI, the presence of ring-shaped calcification corresponding to ring-shaped hypointensity on T2WI was assessed.

### Statistical analysis

All statistical analyses were performed using the Statistical Package for the Social Sciences version 24.0 (IBM Corp.) or EZR (Saitama Medical Center, Jichi Medical University). Tukey’s post hoc test or Welch’s ANOVA with Games–Howell post hoc test was used to compare quantitative measurements (age, maximum diameter, SIR, ADC value, and diameter and maximum thickness of ring-shaped hypointensity on T2WI) between the three pathologies. Fisher’s exact test was performed to compare qualitative assessment parameters (gender, internal uniformity, predominant SI, heterogeneity, hypointensity on T2WI, ring-shaped hypointensity on T2WI, hyperintensity on T1WI, tumor laterality, unenhanced area, tumor margin, extrathyroidal extension, tracheal invasion, esophageal invasion, laryngeal invasion, vascular invasion, venous thrombosis, venous tumor thrombosis, lymphadenopathy, calcification, number and shape of calcification, and ring-shaped calcification corresponding to ring-shaped hypointensity on T2WI) between the three pathologies. When a two-sided *p* value < 0.05 of the test for the three groups was observed, we concluded that there was a difference in the frequency between the groups. Post hoc pairwise comparisons were performed only if the Fisher’s exact test for the three groups was statistically significant. *p* values were corrected according to the Bonferroni method for pairwise comparisons. Interobserver variability in qualitative assessments was evaluated using Kappa statistic. A Kappa value of ≤ 0.20 was interpreted as slight agreement, 0.21–0.40 as fair agreement, 0.41–0.60 as moderate agreement, 0.61–0.80 as substantial agreement, and ≥ 0.81 as almost perfect agreement.

## Results

### Patients

Table 1 summarizes the characteristics of the patients included in this study. The present study enrolled 14 patients with ATCs (age range, 46–83 years; mean age, 71.0 years; 10 men and 4 women), 8 patients with PDTCs (age range, 9–82 years; mean age, 57.4 years; 2 men and 6 women), and 57 patients with PTCs (age range, 6–87 years; mean age, 57.4 years; 23 men and 34 women). The age was significantly higher in ATCs than in PTCs (*p* = 0.047). There was no significant difference in the gender (*p* = 0.059) among patients with ATC, PDTC, and PTC.

**Table 1:** Patient characteristics

Characteristics	ATC	PDTC	PTC
Number of patients	14	8	57
Gender (male:female)	10: 4	2: 6	23: 34
Age (year)			
Range	46–83	9–82	6–87
Mean	71.0	57.4	57.4

*ATC* Anaplastic thyroid carcinoma, *PDTC* Poorly differentiated thyroid carcinoma, *PTC* Papillary thyroid carcinoma

### Imaging findings

**Fig. 1:**
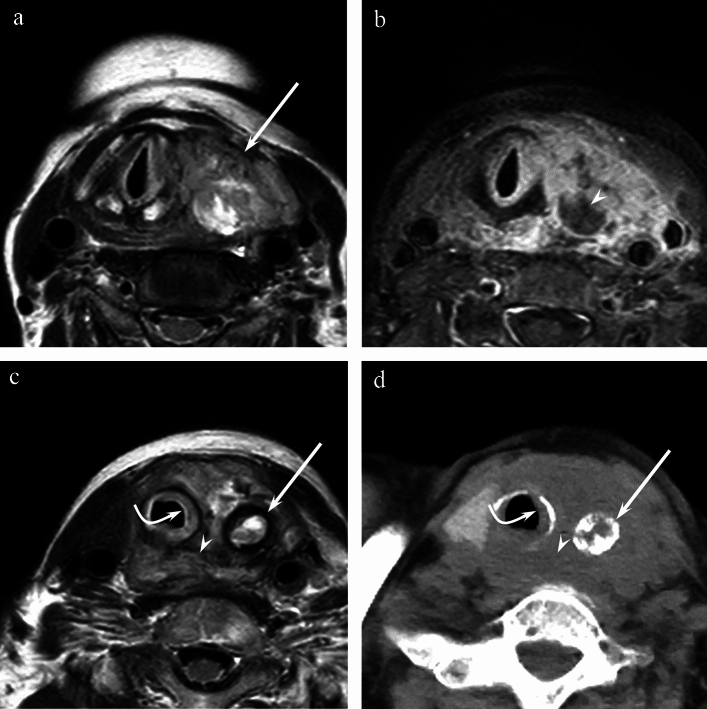
A 79-year-old woman with anaplastic thyroid carcinoma of the left lobe. **a** T2-weighted image (TR/TE, 4620/100 ms) shows a heterogeneously hyperintense mass with an ill-defined margin (arrow). **b** Fat-suppressed contrast-enhanced T1-weighted image (TR/TE, 656/18 ms) shows heterogeneous enhancement with unenhanced areas (arrowhead). **c** T2-weighted image shows intratumoral ring-shaped hypointensity (arrow). Tracheal (curved arrow) and esophageal (arrowhead) invasions are demonstrated. **d** Unenhanced CT image shows intratumoral ring-shaped calcification (arrow) corresponding to the ring-shaped hypointensity on T2WI. The tumor shows an ill-defined margin, tracheal invasion (curved arrow), and esophageal invasion (arrowhead)

**Fig. 2:**
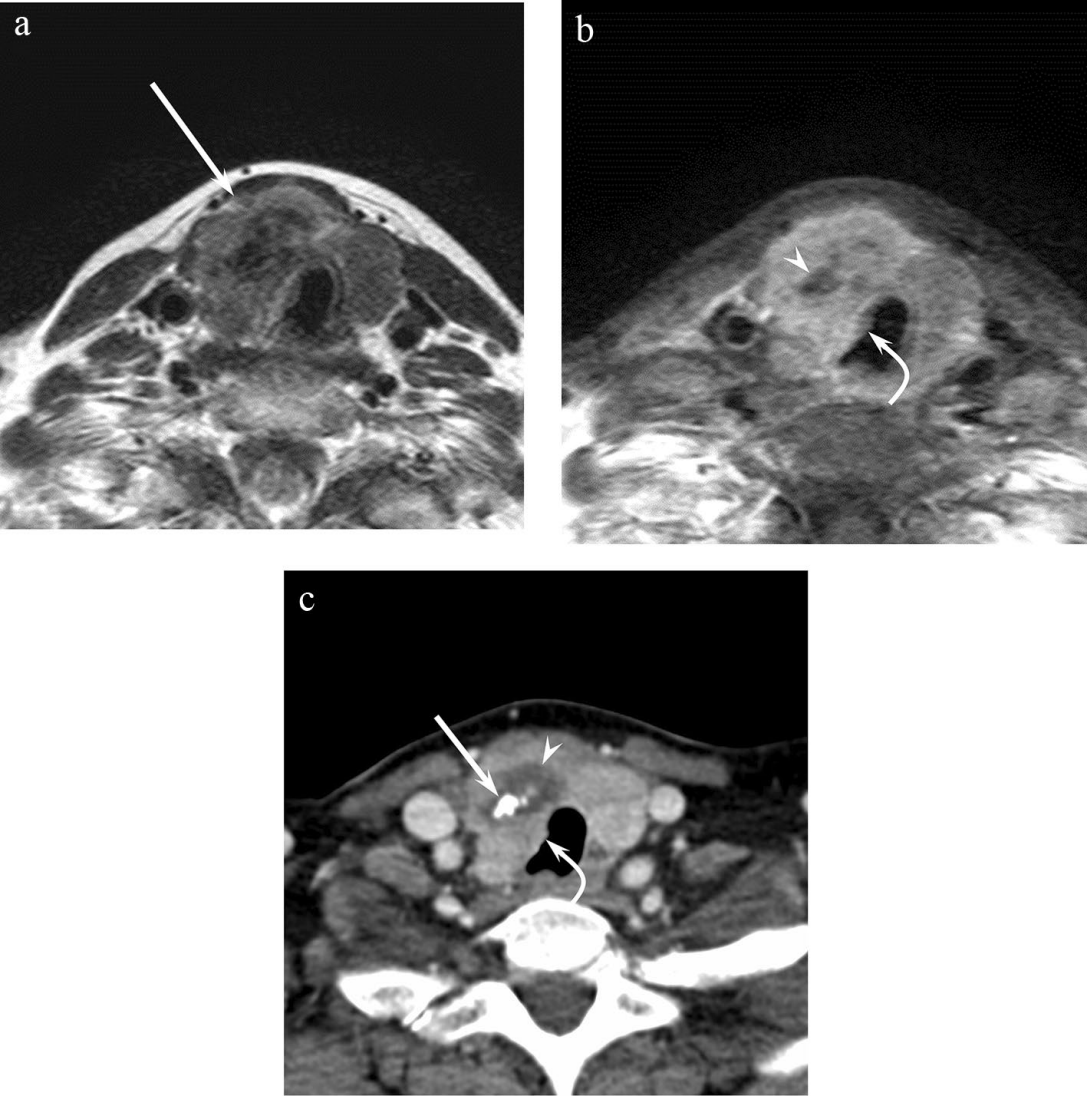
A 64-year-old woman with poorly differentiated thyroid carcinoma of the right lobe. **a** T2-weighted image (TR/TE, 5710/90 ms) shows a heterogeneously mixed hyper- and hypointense mass (arrow). **b** Fat-suppressed contrast-enhanced T1-weighted image (TR/TE, 632/15 ms) shows heterogeneous enhancement with unenhanced areas (arrowhead). The tumor shows an ill-defined margin and tracheal invasion (curved arrow). **c** Contrast-enhanced CT image shows intratumoral nodular calcification (arrow) and unenhanced area (arrowhead). The tumor shows an ill-defined margin and tracheal invasion (curved arrow)

**Fig. 3:**
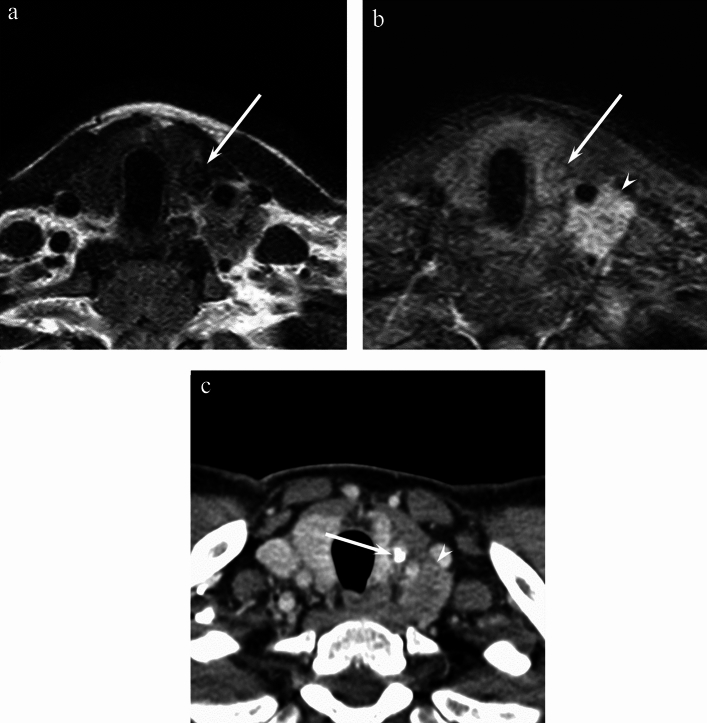
A 38-year-old man with papillary thyroid carcinoma of the left lobe. **a** T2-weighted image (TR/TE, 3456/90 ms) shows a hypointense mass (arrow). **b** Fat-suppressed contrast-enhanced T1-weighted image (TR/TE, 675/17 ms) shows mild enhancement (arrow). The tumor shows a well-defined margin without adjacent organ invasion but left lower jugular lymph node metastasis (arrowhead) is observed. **c** Contrast-enhanced CT image shows intratumoral nodular calcification (arrow) and left lower jugular lymph node metastasis (arrowhead)

Table 2 summarizes the quantitative assessments. The maximum diameter was significantly larger in ATCs and PDTCs than in PTCs (65.2 ± 18.6 mm and 38.4 ± 23.8 mm vs. 26.0 ± 18.8 mm, *p* = 0.000) (Figs. 1, 2 and 3). The SIR of the solid components on T2WI was significantly higher in ATCs than in PTCs (1.13 ± 0.15 vs. 0.89 ± 0.38, *p* = 0.042) (Figs. 1a and 3a). Furthermore, no significant differences were observed in the SIR of the solid components on T1WI (*p* = 0.140) and FS CE-T1WI (*p* = 0.344) and the ADC value of the solid components (*p* = 0.114) between the three pathologies.

**Table 2:** Quantitative assessments of MRI findings

	ATC (*n* = 14)	PDTC (*n* = 8)	PTC (*n* = 57)	*p* value
Maximum diameter (mm)*	65.2 ± 18.6^b^	38.4 ± 23.8^c^	26.0 ± 18.8^bc^	0.000†
SIR of solid component				
T2WI*	1.13 ± 0.15^b^	1.01 ± 0.19	0.89 ± 0.38^b^	0.042†
T1WI*	0.87 ± 0.13	1.00 ± 0.13	0.91 ± 0.16	0.140
FS CE-T1WI*	1.84 ± 0.39	1.52 ± 0.24	1.62 ± 0.45	0.344
ADC of solid component	*n* = 11	*n* = 7	*n* = 50	
Mean ADC (× 10^−3^ mm^2^/s)*	0.84 ± 0.17	1.06 ± 0.26	1.10 ± 0.41	0.114
Intratumoral ring-shaped hypointensity on T2WI	*n* = 9	*n* = 1	*n* = 8	
Diameter (mm)*	33.1 ± 17.4	18	17.7 ± 15.1	0.139
Maximum thickness (mm)*	5.3 ± 5.2	3	2.5 ± 1.0	0.262

*ATC* Anaplastic thyroid carcinoma, *PDTC* Poorly differentiated thyroid carcinoma, *PTC* Papillary thyroid carcinoma, *SIR* Signal intensity ratio, *T2WIs* T2-weighted images, *T1WIs* T1-weighted images, *FS CE-T1WIs *Fat-suppressed contrast-enhanced T1-weighted images, *ADC* Apparent diffusion coefficient

*Data are mean ± standard deviation

^†^*p* < 0.05 between ATCs, PDTCs, and PTCs

^a^*p* < 0.05 between ATCs and PDTCs, ^b^*p* < 0.05 between ATCs and PTCs, ^c^*p* < 0.05 between PDTCs and PTCs

Table 3 summarizes the qualitative assessments of each sequence. The predominant SI of the solid components on T2WI was found to exhibit hyperintensity relative to the spinal cord in ATCs more frequently than in PTCs (71% vs. 30%, *p* = 0.000), whereas hypointensity was more frequent in PTCs than in ATCs and PDTCs (60% vs. 0% and 13%, *p* = 0.000) (Figs. 1a, 3a). Intratumoral ring-shaped hypointensity on T2WI was more frequently observed in ATCs than in PDTCs and PTCs (64% vs. 13% and 18%, *p* = 0.002) (Fig. 1c). Among 9 ATCs, 1 PDTC, and 10 PTCs with intratumoral ring-shaped hypointensity on T2WI, 3 ATCs and 2 PTCs had aggregated and multiloculated rings, whereas the remaining had single rings. Multifocal ring-shaped hypointensities on T2WI were not observed. No significant differences were observed in heterogeneity (T2WI: *p* = 0.055, T1WI: *p* = 0.075, FS CE-T1WI: *p* = 0.174), hypointensity on T2WI (*p* = 0.050), predominant SI on T1WI (*p* = 0.468), hyperintensity on T1WI (*p* = 0.438), and unenhanced area on FS CE-T1WI (*p* = 0.103) between the three pathologies.

**Table 3:** Qualitative assessments of MRI findings

	ATC (*n* = 14)	PDTC (*n* = 8)	PTC (*n* = 57)	*p* value
T2WI				
Heterogeneity	14 (100%)	7 (88%)	42 (74%)	0.055
Predominant SI of solid component				
Hyperintensity	10 (71%)^b^	3 (38%)	17 (30%)^b^	0.000†
Isointensity	4 (29%)	4 (50%)	6 (11%)	
Hypointensity	0 (0%)^b^	1 (13%)^c^	34 (60%)^bc^	
Presence of hypointensity	12 (86%)	3 (38%)	34 (60%)	0.050
Intratumoral ring-shaped hypointensity	9 (64%)^ab^	1 (13%)^a^	10 (18%)^b^	0.002†
T1WI				
Heterogeneity	12 (86%)	4 (50%)	31 (54%)	0.075
Predominant SI of solid component				
Hyperintensity	0 (0%)	2 (38%)	5 (9%)	0.468
Isointensity	8 (57%)	3 (38%)	28 (49%)	
Hypointensity	6 (43%)	3 (38%)	24 (42%)	
Presence of hyperintensity	3 (21%)	0 (0%)	13 (23%)	0.438
FS CE-T1WI	*n* = 8	*n* = 5	*n* = 33	
Heterogeneity	8 (100%)	3 (60%)	26 (79%)	0.174
Unenhanced area	8 (100%)	3 (60%)	21 (64%)	0.103

*ATC* Anaplastic thyroid carcinoma, *PDTC* Poorly differentiated thyroid carcinoma, *PTC* Papillary thyroid carcinoma, *T2WIs* T2-weighted images, *T1WIs* T1-weighted images, *FS CE-T1WIs* Fat-suppressed contrast-enhanced T1-weighted images

^†^*p* < 0.05 between ATCs, PDTCs, and PTCs

^a^*p* < 0.05 between ATCs and PDTCs, ^b^*p* < 0.05 between ATCs and PTCs, ^c^*p* < 0.05 between PDTCs and PTCs

Table 4 summarizes the qualitative assessments of tumor extension. An ill-defined margin was more frequently observed in ATCs and PDTCs than in PTCs (93% and 63% vs. 25%, *p* = 0.000) (Fig. 1a). In addition, extrathyroidal extension (*p* = 0.000), tracheal invasion (*p* = 0.000), esophageal invasion (*p* = 0.006), vascular invasion (*p* = 0.001), and venous thrombosis (*p* = 0.039) were more frequently observed in ATCs than in PTCs (Fig. 1c). Esophageal invasion (*p* = 0.006), vascular invasion (*p* = 0.001), and venous tumor thrombosis (*p* = 0.010) were more frequently observed in PDTCs than in PTCs. There were no significant differences in bilaterality (*p* = 0.295), laryngeal invasion (*p* = 0.283), and lymphadenopathy (*p* = 0.815) between the three pathologies.

**Table 4:** Qualitative assessments of tumor extension

	ATC (*n* = 14)	PDTC (*n* = 8)	PTC (*n* = 57)	*p* value
Bilaterality	5 (36%)	4 (50%)	15 (26%)	0.295
Ill-defined tumor margin	13 (93%)^b^	5 (63%)^c^	14 (25%)^bc^	0.000†
Extrathyroidal extension	13 (93%)^b^	5 (63%)	19 (33%)^b^	0.000†
Tracheal invasion	8 (57%)^b^	4 (50%)^c^	7 (12%)^bc^	0.000†
Esophageal invasion	6 (43%)^ab^	1 (13%)^a^	5 (9%)^b^	0.006†
Laryngeal invasion	2 (14%)	0 (0%)	3 (5%)	0.283
Vascular invasion	3 (21%)^b^	3 (38%)^c^	1 (2%)^bc^	0.001†
Venous thrombosis	2 (14%)^b^	0 (0%)	0 (0%)^b^	0.039†
Venous tumor thrombosis	1 (7%)	2 (25%)^c^	0 (0%)^c^	0.010†
Lymphadenopathy	9 (64%)	4 (50%)	32 (56%)	0.815

*ATC* Anaplastic thyroid carcinoma, *PDTC* Poorly differentiated thyroid carcinoma, *PTC* Papillary thyroid carcinoma

^†^*p* < 0.05 between ATCs, PDTCs, and PTCs

^a^*p* < 0.05 between ATCs and PDTCs, ^b^*p* < 0.05 between ATCs and PTCs, ^c^*p* < 0.05 between PDTCs and PTCs

Table 5 summarizes the CT findings regarding calcification. Calcification within the tumor was observed in 12 ATCs (86%), 3 PDTCs (38%), and 33 PTCs (58%). No significant differences were observed in the presence (*p* = 0.059), number (*p* = 0.637), nodular shape (*p* = 0.876), and ring shape (*p* = 0.483) of calcification between the three pathologies. Ring-shaped calcification corresponding to ring-shaped hypointensity on T2WI (Fig. 1c and d) was observed in 5 of 9 (56%) ATCs, 1 of 1 (100%) PDTCs, and 8 of 10 (80%) PTCs (*p* = 0.545) (Fig. 1d).

**Table 5:** CT findings regarding calcification

	ATC (*n* = 14)	PDTC (*n* = 8)	PTC (*n* = 57)	*p* value
Calcification	12 (86%)	3 (38%)	33 (58%)	0.059
Number of calcification	(*n* = 12)	(*n* = 3)	(*n* = 33)	0.637
Single	8 (67%)	3 (100%)	25 (76%)	
Multiple	4 (33%)	0 (0%)	8 (24%)	
Shape of calcification	(*n* = 12)	(*n* = 3)	(*n* = 33)	
Nodular	7 (58%)	2 (67%)	22 (67%)	0.876
Ring	7 (58%)	1 (33%)	13 (39%)	0.483
Ring-shaped calcification corresponding to ring-shaped hypointensity on T2WI	5 (56%) (*n* = 9)	1 (100%) (*n* = 1)	8 (80%) (*n* = 10)	0.545

*ATC* Anaplastic thyroid carcinoma, *PDTC* Poorly differentiated thyroid carcinoma, *PTC* Papillary thyroid carcinoma, *T2WIs* T2-weighted images

The Kappa values for the two observers exhibited fair agreement regarding the evaluation of predominant SI on T1WI (0.29) and moderate agreement regarding the evaluation of bilaterality, ill-defined tumor margin, heterogeneity on T2WI and T1WI, predominant SI on T2WI, and hyperintensity on T1WI (0.46–0.59). A substantial or almost perfect agreement was observed with respect to other assessments (0.65–1.00).

## Discussion

Compared with PTCs, our study showed that parameters including a larger maximum diameter, higher SI on T2WI, ring-shaped hypointensity on T2WI, ill-defined margin, extrathyroidal extension, tracheal invasion, esophageal invasion, vascular invasion, and venous thrombosis were characteristics of ATCs. Furthermore, a larger maximum diameter, ill-defined margin, tracheal invasion, vascular invasion, and venous tumor thrombosis were characteristics of PDTCs compared with PTCs. Meanwhile, the hypointensity of the solid components on T2WI was characteristic of PTCs compared with ATCs and PDTCs.

In this study, the maximum diameter was significantly larger in ATCs and PDTCs than in PTCs. Although previous CT studies reported various tumor sizes of thyroid cancer (ATCs/PDTCs: 61 mm and PTCs: 45 mm [9]; ATCs: 46 mm and PTCs: 39 mm [7]), the consistent tendency for ATC/PDTC to be larger than PTC was undoubtedly observed because ATCs are clinically characterized by rapid progression with large, hard, and painful neck masses.

On T2WI, the SIR of the solid components to the spinal cord was quantitatively higher in ATCs than in PTCs. In the qualitative assessment of predominant SI on T2WI, hyperintensity was more frequently observed in ATCs than in PTCs, whereas hypointensity was more frequent in PTCs than in ATCs and PDTCs. According to previous studies, intratumoral necrosis is more frequent in ATCs/PDTCs than in PTCs [7, 9], thereby suggesting that liquefactive necrosis and cystic degeneration might contribute to hyperintensity on T2WI. Meanwhile, PTCs usually contain some amount of colloid in the background [10], indicating that viscous colloid might contribute to the hypointensity on T2WI. Reflecting on these histological elements, the SI of solid components on T2WI would tend to be higher in ATCs and lower in PTCs.

Intratumoral ring-shaped hypointensity on T2WI was more frequently observed in ATCs than in PDTCs and PTCs. Our investigation of CT images revealed that ring-shaped calcification corresponding to ring-shaped hypointensity on T2WI was observed in 5 of 9 (56%) ATCs, 1 of 1 (100%) PDTCs, and 8 of 10 (80%) PTCs. Therefore, it can be inferred that ring-shaped hypointensity is caused by ring-shaped calcification or hemosiderin deposition following hemorrhage.

An ill-defined margin was more frequently observed in ATCs and PDTCs than in PTCs, which is consistent with the findings of previous CT studies [7, 9]. ATC is one of the most aggressive types of cancer in humans and is often accompanied by huge inflammatory cell infiltration [11]. Thus, an ill-defined margin in ATCs would reflect invasive tumor growth or peritumoral inflammation.

Extrathyroidal extension, tracheal invasion, esophageal invasion, vascular invasion, and venous thrombosis were more frequently observed in ATCs than in PTCs. Previous CT studies also reported that adjacent organ invasion was more frequently observed in ATCs/PDTCs than in PTCs [7, 9]. More specifically, local invasion into the surrounding structures such as muscles, trachea, esophagus, laryngeal nerve, and larynx was found in approximately 70% of patients with ATCs [12]. The aggressive nature of ATCs would therefore result in a high prevalence of adjacent organ invasion.

The ADC value calculated from DWI is a useful parameter for differentiating malignant from benign thyroid nodules [13–16]. One previous study showed that the mean ADC values of malignant and benign solitary thyroid nodules were 0.73 ± 0.19 × 10^−3^ mm^2^/s and 1.8 ± 0.27 × 10^−3^ mm^2^/s, respectively [13]. Another study showed that the ADC value of thyroid gland malignancies was 0.70 ± 0.31 × 10^−3^ mm^2^/s, while that of benign nodules was 2.75 ± 0.60 × 10^−3^ mm^2^/s [15]. However, no study has shown the differences in the ADC values of each histological subtype of thyroid cancer. In the present study, the ADC values of ATCs tended to be lower than those of PDTCs and PTCs; however, no significant differences were observed in the ADC values of the solid components between the three pathologies. Owing to the fact that the cell density of ATCs is relatively high, its ADC value must be low. However, liquefactive necrosis and cystic degeneration within ATCs may result in an increase in the ADC values.

ATCs are characterized by large necrotic and hemorrhagic masses. Hypercellularity, large necrotic foci of necrosis, marked invasiveness, and angiotropism with a tendency to infiltrate medium-sized veins and arteries are common features of ATCs [11], while cystic degeneration is often observed in ATCs [8], similar to other thyroid masses. Thyroid cystic changes may result from infarction and other destructive processes such as hemorrhage into preexisting follicles or cavities. Infarction may occur after the growth of the nodules, and repeated hemorrhage can cause expansion of thyroid follicles [17]. ATCs with marked tumor expansion and hemorrhage are more likely to undergo cystic changes compared with other thyroid masses.

PDTCs are defined as malignant follicular cell-derived tumors with intermediate morphological features and biological behavior between WDTCs and ATCs. Macroscopically, PDTCs exhibit invasive growth, but occasionally have fibrous capsules, and histologically, the presence of capsular invasion, vessel invasion, distant metastasis, poorly differentiated components with solid/trabecular/insular growth patterns, and coagulation necrosis are important for the diagnosis of PDTCs [2]. In this study, the larger maximum diameter, ill-defined margin, tracheal invasion, vascular invasion, and venous tumor thrombosis were found to be characteristics of PDTCs compared with PTCs, whereas intratumoral ring-shaped hypointensity on T2WI and esophageal invasion were less frequently observed in PDTCs than in ATCs. These results suggest that the MRI findings of PDTCs are intermediate between those of ATCs and PTCs.

Our study has several limitations. First, this was a single-center retrospective analysis, and the number of patients enrolled was relatively small. Second, the MRI findings were acquired using three different MRI scanners due to the retrospective nature of the present analysis. Third, not all patients underwent diffusion-weighted and contrast-enhanced MRI. However, we were able to delineate significant characteristic MRI features for different tumor subtypes. Fourth, 8 of 14 patients with ATC did not undergo removal surgery because of their advanced local invasion. These patients were treated by radiotherapy and/or molecular targeted drugs after surgical biopsy. Therefore, histological investigations regarding extrathyroidal extension and ring-shaped hypointensity on T2WI could not be performed.

In conclusion, higher SI and ring-shaped hypointensity on T2WI were characteristic MRI findings of ATCs compared with PTCs. Larger size, an ill-defined margin, and adjacent organ invasion were morphological features of ATCs compared with PTCs. PTCs predominantly showed hypointensity of the solid components on T2WI compared with ATCs and PDTCs. Finally, PDTCs tended to have intermediate MRI features between ATCs and PTCs.
